# Item development and pre-testing of an Osteoarthritis Conceptualisation Questionnaire to assess knowledge and beliefs in people with knee pain

**DOI:** 10.1371/journal.pone.0286114

**Published:** 2023-09-29

**Authors:** Brian W. Pulling, Felicity A. Braithwaite, David S. Butler, Anna R. Vogelzang, G. Lorimer Moseley, Mark J. Catley, Carolyn M. Murray, Tasha R. Stanton

**Affiliations:** 1 IIMPACT in Health, University of South Australia, Kaurna Country, Adelaide, South Australia, Australia; 2 Persistent Pain Research Group, Hopwood Centre for Neurobiology, South Australia Health and Medical Research Institute (SAHMRI), Adelaide, South Australia, Australia; Duervation, AUSTRIA

## Abstract

Many people with osteoarthritis hold beliefs that physical activity is unhelpful or dangerous for their joints, despite high-level evidence suggesting otherwise. Recent advances in scientific understanding of osteoarthritis have led to new treatments that target an individual’s understanding both of their condition and the importance of best-practice management strategies, such as physical activity. Conceptual change has been proposed as an important mechanism by which cognitive interventions, such as pain science education, may reduce pain and improve function. There are currently no specific assessments of osteoarthritis conceptualisation to determine the effectiveness of cognitive interventions in effecting conceptual change in people with knee osteoarthritis. Therefore, we aimed to develop an item bank, as the first phase of developing a questionnaire to assess people’s conceptualisations about their knee osteoarthritis and the role of physical activity in managing their osteoarthritis. Using a guideline-informed mixed method design, a panel of experts identified domains relevant to conceptualisation about knee osteoarthritis and physical activity (knowledge, beliefs, understanding) based upon available evidence. The panel created 33 provisional items. Qualitative and quantitative pretesting were used to explore how people with knee osteoarthritis understood the provisional items. Eighteen people with knee osteoarthritis completed cognitive interviews about their comprehension of the wording/grammar of each provisional item. The provisional item bank was field tested with 100 people with knee osteoarthritis. Readability was adequate with a Flesch reading ease score of 57.7. Although 14.7% used the ‘Strongly agree’ response option, only 3.4% of responses used the ‘Strongly disagree’ option, suggesting possible response bias. Predictive quality testing identified relevant modifications to the questionnaire instructions. The panel of experts appraised the qualitative data to assess whether and how items should be modified to address the problems identified, resulting in a final item bank of 45 items that can be evaluated for psychometric properties in future research.

## Introduction

Osteoarthritis (OA) affects 240 million people globally [1]. Approximately 9.6% of men and 18% of women aged over 60 years worldwide have symptomatic osteoarthritis [1]. Osteoarthritis has significant personal and economic burden, and results in diminished independence and quality of life for older adults [2–4]. Treatment guideline recommendations advise conservative care to improve function and quality of life through exercise and management of psychosocial factors that impact symptoms [5, 6].

Despite these recommendations, qualitative research shows that many people with osteoarthritis hold misconceptions that physical activity worsens the progression of osteoarthritis [7]. These beliefs may stem from a historical interpretation of osteoarthritis through a biomedical model, wherein physical activity is believed to contribute to joint damage (‘wear and tear’), and where joint degeneration (‘bone on bone’) is seen as the primary driver of pain [7]. However, advances in scientific understanding of osteoarthritis pathogenesis, including the presence of systemic, low level inflammation [8–10], non-joint related contributing factors [2, 11], as well as positive findings for treatment response to exercise and overall prognosis (versus progressive decline) [12, 13], suggest that a purely biomedical interpretation of this condition is not accurate, nor sufficient for treatment [14]. For instance, exercise has been shown to improve osteoarthritic pain, even in those with end-stage arthritis who are awaiting joint replacement [15], with high-level evidence supporting the safety of undertaking exercise for people with osteoarthritis [16]. Misconceptions about osteoarthritis and negative beliefs surrounding exercise reduce participation in physical activity, particularly when it is initially pain-provoking [17]. Consequently, these unhelpful conceptualisations may be a critical barrier to activity engagement.

Interventions such as pain science education (also called ‘Explaining pain’) [18] have been developed to directly target these unhelpful conceptualisations, which are common for those with osteoarthritis [7, 17, 19, 20]. Pain science education aims to help people to re-conceptualise pain, shifting from viewing pain as solely a marker of tissue damage to that of the need to protect the body from real or perceived threat [21]. Such re-conceptualisation involves learning about: i) the biopsychosocial underpinnings of pain; ii) the wide-spread adaptations within the central nervous system occurring in persisting pain states and resulting in enhanced sensitivity; and iii) that multiple factors influence pain and inflammation.

Application of conceptual change theory has been recommended for optimising clinical interventions such as pain science education [21, 22]. Conceptual framework theory [23] offers a psychological perspective on how humans learn and adapt. Briefly, this theory offers an explanation for how humans take on new information, integrate it into their experience, and use this constructed ‘framework’ to conceptualise their world [24]. However, most of the experimental work in this area has focused on educational and developmental psychology [25], and on facilitating conceptual change in science and maths education [26–28].

A comprehensive body of work has evaluated pain science education [29–35], but formal application and testing of conceptual change theory has not yet been undertaken. The mechanism of action for pain science education interventions is thought to be due to conceptual change [21], with changes in pain knowledge as a result of the intervention thought to mediate the resultant clinical outcome. If, as proposed elsewhere, knowledge is only one aspect of a person’s conceptual framework [23], then there is an opportunity to develop more nuanced assessments of conceptual frameworks relevant to pain science education interventions. Further, studies of pain science education-based interventions typically show small to moderate effect sizes for clinical outcomes [32, 33], thus assessment of conceptual frameworks (and conceptual change) may have potential to improve this intervention by better understanding how it works. If we are to test this idea in the field of osteoarthritis pain, we first need a valid assessment for how people understand osteoarthritis and physical activity.

We aimed to fill this critical gap by using a guideline recommended [36] three-phase approach to develop and validate a questionnaire about conceptual frameworks for knee osteoarthritis: (1) develop a bank of items about knee osteoarthritis and physical activity conceptualisation; (2) develop a questionnaire and evaluate its psychometric properties; and (3) evaluate the validity of the questionnaire. The current study presents the first of these phases. As discussed elsewhere [37], the process of item development and pre-testing is often poorly or under-reported. With this in mind, we use best-practice methods for conducting and reporting cognitive interviews for the first phase of scale development.

## Methods

**Fig 1:**
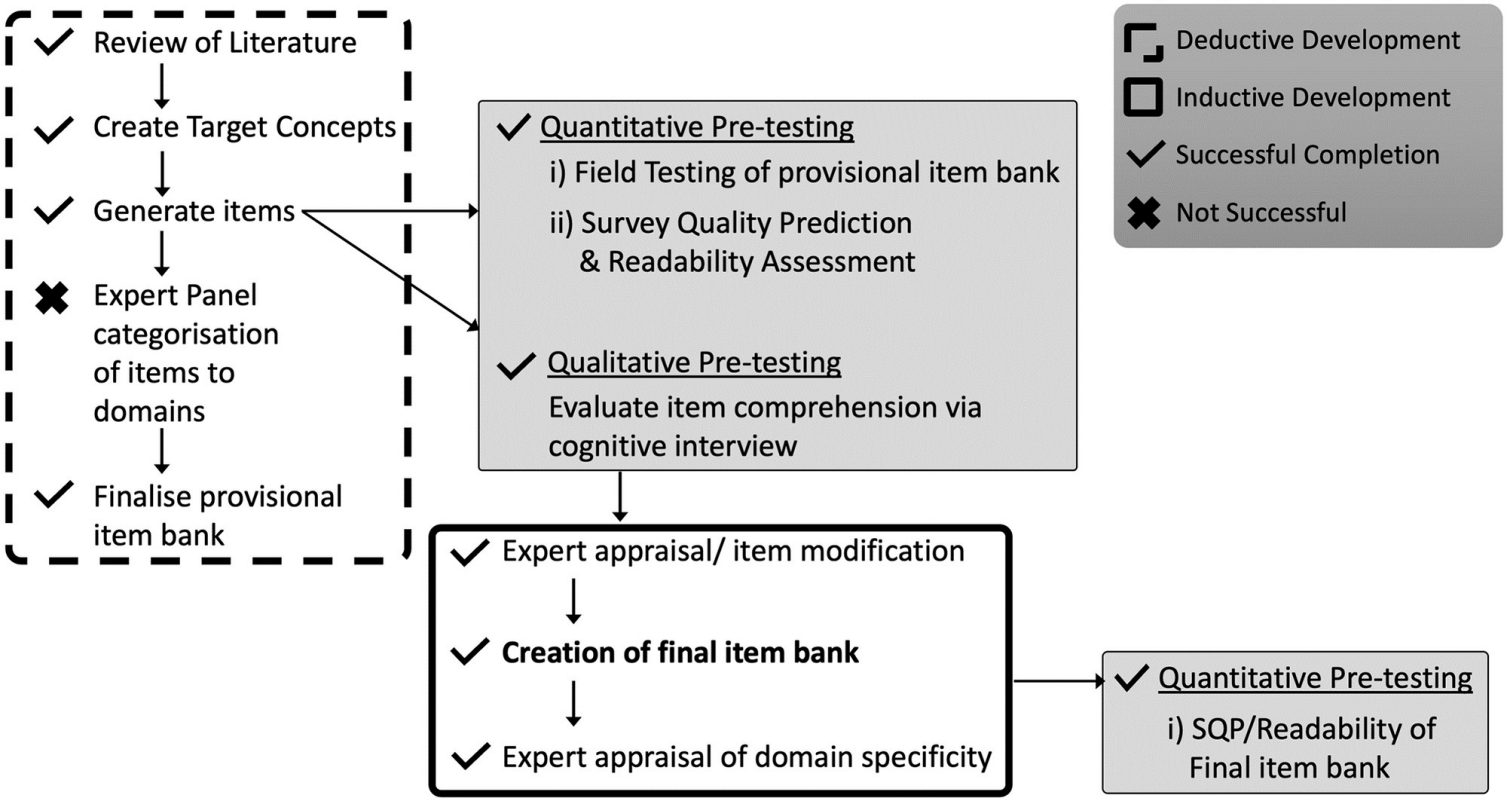
Overview of the item development process for phase one.

We conducted a mixed method item development study focused on domain identification and item generation by an expert panel, with pretesting of provisional items (Fig 1). Pre-testing involved qualitative interviews and quantitative evaluation to explore how people with knee osteoarthritis understood these items. Pre-testing data were used to make modifications to items with the aim to improve item comprehension for the target population of people with knee osteoarthritis. This study used an iterative and reflective approach, whereby the expert panel utilised both deductive and inductive methods to develop, revise, and redevelop provisional items. The study protocol was registered and locked on the Open Science Framework prior to commencement of quantitative analysis (https://osf.io/6xavz/), as per recommendations in the pain field [38]. Project updates and protocols for Phases two and three will be registered on the Open Science Framework (https://osf.io/7b6gj/).

### Ethics

The study was approved by the University of South Australia Human Research Ethics Committee (ID202377). Participants were informed about the nature and purpose of the study and provided written informed consent. Participants were reimbursed AUD$20 for their participation.

### Domain identification & item generation

Domain identification and item generation were conducted by a panel of experts in knee osteoarthritis research, pain science education, and clinical practice (Table 1).

**Table 1:** Expert panel credentials and experience.

Name	Professional Credentials	Experience
Brian W. Pulling	BSc, MRes	PhD Candidate–Health Sciences, UniSA; Tutor in pain science, research methods, and statistics coursework; former clinical research coordinator at Boston Children’s Hospital/Harvard Medical School.
Felicity A. Braithwaite	PhD, BPT(Hons)	Postdoctoral Fellow, UniSA. She is the coordinator of a multi-site randomised controlled trial on osteoarthritis and physical activity and recipient of the John Stuart Colville Fellowship (Arthritis Foundation of South Australia).
G. Lorimer Moseley	DSc, PhD, FAAHMS, FACP, HonFFPMANZCA, HonMAPA, BAppSc(Phty)(Hons)	Bradley Distinguished Professor, Chair in Physiotherapy, Professor of Clinical Neurosciences, Director of IIMPACT in Health at UniSA. He has published numerous papers and seven books on pain science & education.
David S. Butler	B.Phty, M.App.Sc, EdD	Adjunct Associate Professor, UniSA. Founder of the Neuro Orthopaedic Institute (NOI Group). He has published numerous papers and eight books on pain science education and health-related conceptual change.
Anna R. Vogelzang	BClinExPhys	MRes Candidate–Health Sciences, UniSA; Accredited Clinical Exercise Physiologist; former research assistant at Neuro Orthopaedic Institute.
Mark C. Catley	PhD, BPT	Lecturer in Physiotherapy–Pain Sciences, UniSA. He has published numerous papers on scale development, most notably the revised Neurophysiology of Pain Questionnaire used in many pain science education trials.
Tasha R. Stanton	BScPT, MScRS, PhD	Associate Professor in Clinical Pain Neuroscience, Osteoarthritis Research Theme Lead for IIMPACT in Health at UniSA. She has published numerous papers on pain in osteoarthritis, and a book on pain education in osteoarthritis. She is Principal Investigator, National Health & Medical Research Council of Australia on a clinical trial of pain science education in osteoarthritis.

Two members of the expert panel (DSB, ARV) conducted an informal literature review to synthesise contemporary osteoarthritis pathogenesis [5, 8, 10, 13, 15, 39–41], and to identify conceptualisations about osteoarthritis and exercise/activity held by people with lived experience of osteoarthritis [6, 7, 13, 14, 20, 42]. Five target concepts were adapted from the *Explain Pain Protectometer* curriculum [43, 44] representing best practice contemporary pain science education [45]. The five target concepts were: 1) learning about knee osteoarthritis is an effective intervention and the basis of treatment; 2) knee osteoarthritis is a mild chronic (and sometimes painful) inflammatory, changeable bodily process that manifests in the knee; 3) pain is a protective process and one of many protective systems and processes in the body; 4) pain is completely dependent on context; 5) healing and change are irresistible forces of nature. It was intended that these five target concepts may constitute five domains that could be validated using a future factor analysis. These target concepts are mapped as a matrix to the literature identified in the informal literature review (Table 2).

**Table 2:** Literature review mapped to target concepts and general misconceptions.

A. Educational Curriculum Target Concepts	Relevant Literature
1) Learning about knee osteoarthritis is an effective intervention and the basis of treatment	[6, 7, 14, 15, 42]
2) Knee osteoarthritis is a mild chronic (and sometimes painful) inflammatory, changeable bodily process that manifests in the knee	[5, 8, 10, 13, 15, 39]
3) Pain is a protective process and one of many protective systems and processes in the body	[8, 10, 39]
4) Pain is completely dependent on context	[20, 40, 41]
5) Healing and change are irresistible forces of nature	[13, 42]
B. Misconceptions about knee osteoarthritis and physical activity	[6, 7, 14, 20]

Initial deductive item generation was informed by the five target concepts. Potential items were discussed iteratively by the panel, with discussion focussed on merit and concern for each item. Considerations included perceived versus intended meaning, linguistic style, literacy and readability, cross-cultural generalisability, relevance, measurement utility, and dimensionality [36, 45]. The panel aimed to create items that were worded as simply as possible, while maintaining accuracy; formal evaluation of readability levels occurred via qualitative testing. The panel voted in real-time on item selection and modification.

**Fig 2:**
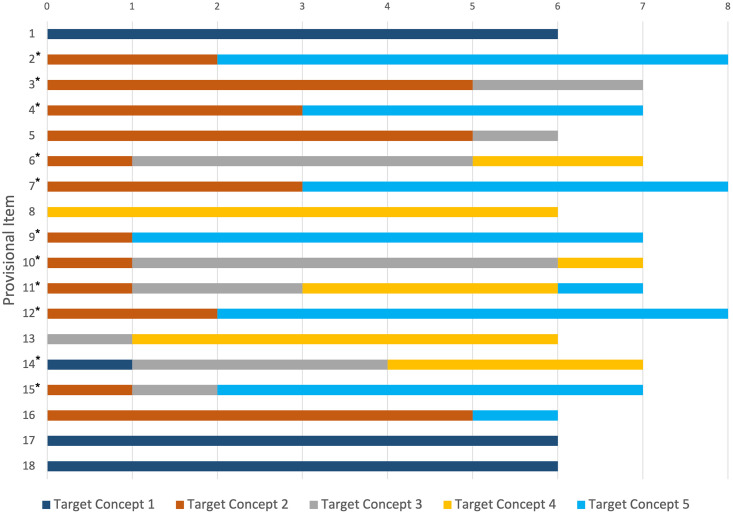
Item-target concept rater-agreement. *Deemed to fit into more than one target concept by at least one assessor.

In order to assess validity of the provisional item bank, we adopted the COSMIN definition for content validity as “the degree to which the content of a health-related patient reported outcome is an adequate reflection of the construct to be measured” [46 p743]. To estimate content validity and domain specificity for provisional items, panel members independently categorised each remaining item into one (or more) of the initial five target concepts for which they felt the item was best suited. Panel agreement (by count and percentage agreement) for these item categorisations was used to evaluate the presence (or absence) of domain specificity (Fig 2). Given that some but not all panel members assigned each item to more than one target concept, no inter-rater reliability assessment was undertaken. Categorisations were also used to stimulate discussion about further item modification and item selection. The provisional questionnaire used a 5-item Likert response scale, anchored by ‘Strongly agree’, ‘Agree’, ‘Neutral’, ‘Disagree’, and ‘Strongly disagree’, however, response distributions were calculated using numeric scores without correction.

### Qualitative pre-testing

Pretesting methods and analysis are reported using the Cognitive Interviewing Reporting Framework (CIRF) [47] and the Consolidated Criteria for Reporting Qualitative Research (COREQ) [48] as appropriate. Per recommendations [36], cognitive interviews [49] were conducted with people who had a clinical diagnosis of knee osteoarthritis to identify problems in wording, jargon, and comprehension of each item. These data were used to estimate content validity in the population of interest, and to improve the quality of item development [36, 45, 49, 50].

#### Eligibility criteria

People who met the NICE guidelines for osteoarthritis diagnosis (knee pain lasting 6 months or longer; aged 50 years or older; morning knee stiffness lasting no more than half an hour or no morning stiffness) [51] were eligible for inclusion. People were not eligible to participate if they met any of the following criteria: knee replacement in their painful knee (if unilateral osteoarthritis); neurological disorder affecting lower limb movement (e.g., multiple sclerosis, stroke); inflammatory arthritis (including rheumatoid arthritis); fibromyalgia or persistent widespread pain; or inability to read and understand English (prohibiting completion of questionnaires and interview) per self-report.

#### Sample size and recruitment

Based on recommendations for pretesting sample size [52–54], we aimed to recruit between 15 and 20 participants with osteoarthritic knee pain from the community for a one-hour interview. Variety in recruitment sources (and their resultant conceptualisations about osteoarthritis and physical activity) was undertaken in pursuit of data triangulation [55]. Community members were invited to participate via posters, social media posts, and an email newsletter distributed by consumer advocacy body Arthritis South Australia, as well as via the recruitment list and pilot participant list of an ongoing randomised controlled trial (RCT) involving people with knee osteoarthritis [44]. The RCT pilot participants had recently completed a contemporary osteoarthritis and pain science education intervention that aimed to change conceptualisation about osteoarthritis and physical activity. The RCT pilot participants were recruited for their unique insight into a practical application of the questionnaire (given they had received an intervention aiming to induce conceptual change), and their potentially updated conceptualisation of knee osteoarthritis and physical activity than that held by people from the general community.

#### Pre-interview assessment

Participants completed an online survey, including demographic questions (date of birth, sex, highest level of completed education), the provisional item bank, as well as the Western Ontario and McMaster Universities Osteoarthritis Index [56], and the brief Fear of Movement scale [57]. While completing the item bank, participants were asked to identify any items with words or phrases that were confusing (yes/no); if they marked ‘yes’, they were asked to type a response explaining what was confusing and why. These reports were used to guide interview probes.

#### Cognitive interview

Following completion of the online survey, interviews were conducted either in-person at the University of South Australia or via telephone or video conferencing (Zoom, San Jose, California, USA) and included questions relating to both wording comprehension and content validity (See S1 and S2 Appendices). All interviews were conducted by author BWP, who was trained in qualitative interview methods by an expert qualitative researcher (CMM), and via published training materials [49, 52, 58–62]. After the first interview, two researchers (FAB and CMM) discussed the process with the interviewer to provide additional reflexivity and skill development in interviewing.

Interviews were semi-structured using a combination of think-aloud interviewing, comprehension/interpretation probing, paraphrasing, general probes, and spontaneous probes [52]. The interviews were structured in three parts. Participants were asked to (1) define a series of words and phrases used in the provisional items; (2) answer a series of questions about each item; (3) answer questions about their perceptions of the purpose of the questionnaire as a whole (as an assessment of face validity). The RCT pilot participants [44] were also asked several additional questions based on their experience in that trial; those data were collected for a separate study and will not be reported on, as per our *a priori* protocol.

In the first stage, participants were asked to provide a definition of what they understood the following terms to mean: osteoarthritis; outcome; pain; inflammation; exercise; physical activity; flare-up; activity; protective system. This stage was included to better understand their perspectives about osteoarthritis for a separate project (registration: https://osf.io/wkgfr/).

The second phase of the interview was structured such that participants responded to a series of tasks and questions for each item from the provisional item bank. The same tasks/questions were repeated for each item. First, participants were asked to use a think-aloud procedure, where they read the item aloud and then articulated their thought process while considering and responding to the item [63]. Next, participants were asked if “they found anything about that statement challenging?”, and this probe was followed up with additional probes (“if yes, what/why”; “can you tell me more about that?”). Lastly, participants were asked to put the statement into their own words.

In the third stage, participants were asked to respond to scripted questions about the questionnaire as a whole (e.g., “What do you think the questionnaire is about?”; “If you are unsure what it is about, what is your best guess?”). Participants were also asked if they had any final thoughts or suggestions for the researchers. Participants did not review the transcripts but clarifying questions were asked by the researcher during the interview.

#### Data analysis: Cognitive interviewing/expert appraisal

We anticipated that many participants may misunderstand the content/meaning of the items, consistent with a naïve conceptualisation of osteoarthritis and physical activity. Therefore, during this phase we only considered problems related to concept comprehension, judgement, or interpretation of content. For instance, a comment that “I don’t understand why inflammation is important” would not be considered a problem with the questionnaire item, because people who have not been exposed to contemporary research findings about the role of inflammation in osteoarthritis would not be expected to understand this connection. Quotations that identified wording and grammar problems with the provisional items were extracted by the interviewer (BWP), with a particular focus on responses to the questions “was anything about that item challenging?” and “were there any words or phrases in that item that were unclear to you?”. The quoted problems specific to each item were compiled and presented to the expert panel, along with a proposed potential solution to each problem. For example, alternative phrasing of the item in line with participant suggestions was provided. Solutions were devised by the interviewer for review by the expert panel.

Expert appraisal was used to interpret the interview data and address problems with the provisional items [36, 49]. The panel discussed each item and its problems in a similar manner as was used to initially develop the provisional items [52]. The interviewer attended the appraisal meetings, to present the problems/participant feedback, but did not contribute any opinion during the panel discussion to avoid biasing the panel. Consistent with recommendations [52, 63] and previous work [64], problems were considered based on their merit as decided by the expert panel, rather than by their frequency or type. The experts were instructed that, pending a unanimous vote, they could maintain the original wording of the item, modify the item, delete the item, or develop new item(s). The experts were also instructed to discuss and reach consensus about the intention and meaning of each item in context of the questionnaire, to contextualise future factor analysis (Phase 2 of scale development). Lastly, in context with the qualitative interview summary findings, the expert panel also considered and discussed the instructions provided to participants prior to completing the items, and whether any changes were required.

### Quantitative pretesting

In parallel with qualitative pretesting, we evaluated the provisional item bank using quantitative field testing, survey quality prediction, and a readability assessment.

#### Field testing

The provisional item bank was administered as part of the baseline assessment in an ongoing RCT; [44] data from a sub-sample of this population was used for the current evaluation. Participants in this trial met the NICE guidelines for osteoarthritis diagnosis [51]. Per guidelines, a minimum sample of 30 participants was sufficient to power for pretesting, however, as the trial was ongoing at the time of protocol registration, we prospectively planned to include all available data at the time of manuscript preparation [53, 65]. *A priori* analysis included the reporting of response distribution, tabulation, the presence of missing data, and floor/ceiling effects. We defined floor/ceiling effects when more than 15% of the population achieve the lowest or highest possible total score, as per previous work [66, 67].

#### Survey Quality Prediction (SQP)

We undertook survey quality prediction using SQP 2.1 (http://sqp.upf.edu/), a tool that extrapolates validity and reliability of a questionnaire from the systematic error and random error present in a questionnaire [68]. This approach uses machine learning to provide estimates of important aspects of reliability and validity prior to survey administration [69]. Survey quality prediction facilitates quantitative pretesting based on the formal and linguistic characteristics of questionnaire instructions and items, resulting in a prediction of questionnaire quality. Predicted quality is calculated as a function of the predicted validity and predicted reliability, whereby validity is the proportion of method error variance in the true score and reliability is the proportion of random error in the observed variance [69]. This predictive algorithm has been developed via meta-analyses of studies that use modified versions of the same questionnaires but with variations in the questionnaire characteristics (e.g. the type of response scale used, the tone of the instructions provided) [69]. These predictions do not provide traditional estimates of reliability or validity, but rather, can offer an insight into how changes to questionnaire characteristics may impact the overall quality of the questionnaire.

#### Readability

Readability was assessed using the Flesch Reading Ease score, which imputes acceptability of a text to various reading levels based on word and sentence complexity [70]. Flesch Reading Ease scores ranging between 50.0 to 60.0 are considered appropriate for a secondary school reading level [70].

### Data handling and management

Study data were collected and managed using REDCap [71, 72] electronic data capture tools hosted at the University of South Australia. Qualitative analyses were conducted using Microsoft Word. Qualitative interviews and expert appraisal meetings were audio recorded and software transcribed (Descript online) with manual review (BWP) to check accuracy of transcription. Quantitative analyses were conducted with Microsoft Excel and SQP 2.1 [69].

## Results

### Domain identification and item generation

The members of expert panel met five times between April and August 2020 to generate, select, and remove items from the provisional item bank. Evaluation of content validity and domain specificity found inconsistent agreement in the categorisation of items to target concepts (70% agreement, ±28%). These findings supported the need for further consideration of domain specificity by the expert panel, following evaluation of face validity of these items by consumers. Thus, without further consideration for the target concepts, deliberation and modification to the remaining items was undertaken based on expert opinion, resulting in 33 provisional items: 20 items about knee osteoarthritis and 13 items referring to physical activity (see S3 Appendix).

### Qualitative pretesting

Eighteen people (10 female, 8 male) with painful knee osteoarthritis were recruited in South Australia, with a mean age of 71.8 (±7.5). Eight were recruited from the RCT ineligibility list (too physically active to be eligible for the trial) [44]. Seven were recruited via posters, social media, and mailing lists. Three participants were recruited from those who had completed the RCT intervention pilot study [44]. Table 3 provides the participant demographic characteristics/clinical measures.

**Table 3:** Pretesting participant characteristics.

Characteristics	Qualitative Pretesting (n = 18)	Quantitative Pretesting (n = 100)
Recruitment location		
South Australia, Australia	18	79
Victoria, Australia	—	21
EPIPHAKnee Trial ineligibility list	8	—
Community posters, social media, mailing lists	7	—
EPIPHAKnee Trial	3	100
Sex (count)		
Female	10	68
Male	8	31
Other	—	1
Age (years)	71.8 (7.5)	66.9 (9.0)
50–59	1	22
60–69	6	43
70–79	9	26
80–89	2	8
90–99	—	1
Highest level of completed education (count)	18	100
Primary school/Did not complete high school	1	12
High school	5	16
Trade, certificate, or diploma	3	25
Bachelor’s degree	4	26
Masters or PhD	5	21
Duration of knee pain (years)	9.5 (11.7)	8.71 (9.79)
Knee pain intensity during previous week (0–10 NRS)	5.4 (1.6)	5.88 (1.49)
Knee pain intensity while walking during previous week (0–10 NRS)	5.3 (1.9)	6.28 (1.67)
Interview duration (minutes)	74.8 (12.2)	—
WOMAC (five-point Likert)	34.3 (13.3)	38.29 (12.21)
BFOM (four-point Likert)	6.8 (2.6)	7.91 (3.65)

Values indicate mean and standard deviation unless otherwise specified.

Western Ontario and McMaster Universities Osteoarthritis Index (WOMAC); Brief Fear of Movement (BFOM)

Participants identified potential problems with all but four items (see S4 Appendix). There were problems with ‘double-negatives’ (e.g., “My age does not determine whether or not I can improve my osteoarthritis”), and so-called ‘double-barrelled’ items that included multiple concepts (e.g., “When osteoarthritis becomes ‘bone-on-bone’ physical activity can no longer help; only surgery can help”).

### Expert appraisal

Expert appraisal was conducted in-person with the original expert panel, across three meetings between September and October 2021. Each meeting lasted approximately 100 minutes. Of 29 items with problems regarding wording comprehension, the expert panel modified 25 items and left four items unchanged because they were determined to not constitute problems with wording comprehension. Two items that participants did not report problems for were changed by the panel. Following the final expert appraisal meeting, the interviewer proposed six additional items to the panel based on information attained from the full cognitive interviews–the experts unanimously agreed that these additional items should be included. In this process, some items were slightly reworded to ensure a roughly equivalent number of positively and negatively loaded items in each domain.

Although some participants (n = 6) reported a preference for the inclusion of an ‘unsure’ response option, this was not adopted, given that scale measurement properties are compromised if too many participants choose this option [73, 74]. The panel instead voted to change the middle anchor of the Likert scale from “neutral” to “neither agree nor disagree” to provide consistent gradation of the scale anchors.

The questionnaire instructions were substantially modified because they were deemed ambiguous and potentially misrepresentative of the objectives for the future questionnaire. The instructions used for the cognitive interviews read: “This questionnaire is not a test of your knowledge about knee osteoarthritis. If you feel like you do not know the answer to a question, please answer the question based on what you believe would be true”. Following expert discussion, the finalised instructions are as follows: “We are interested in your thoughts about osteoarthritis, as well as physical activity. Please indicate how much you agree or disagree with each of the following statements by using the following scale.” These changes are further described below in terms of survey quality prediction.

### Osteoarthritis Conceptualisation Questionnaire

The final result of this project (Phase 1) is an item bank consisting of 45 items for further evaluation in the development of a new questionnaire, tentatively titled the **O**steo**a**rthritis **C**onceptualisation **Questionnaire** (Table 4).

**Table 4:** Item bank for continued development of the Osteoarthritis Conceptualisation Questionnaire.

Item Wording	Domain
Doing more than my usual amount of activity will damage my joint*	Activity and damage
My knee with osteoarthritis should be rested as much as possible*	Activity and damage
Gradually increasing my activity level over time will not damage my joint	Activity and damage
Exercising wears out my knee joint*	Activity and damage
The outcome for my knee is unchangeable*	Changeability
My osteoarthritis will get worse over time no matter what I do*	Changeability
Because my osteoarthritis involves more than my knee, I can change the outcome for my knee	Changeability
Because my osteoarthritis involves more than my joint, there are many different ways I can improve my symptoms	Changeability
“Bone-on-bone” is an accurate descriptor of my osteoarthritis*	Definition of osteoarthritis
“Wear-and-tear” is an accurate descriptor of my osteoarthritis*	Definition of osteoarthritis
Exercise can reduce joint inflammation in my osteoarthritis	Exercise
Knowledge about osteoarthritis can help me to exercise in a safe way	Exercise
Exercise can make the joint surfaces in my knee healthier	Exercise
Understanding how exercise works will influence my osteoarthritis	Exercise
My beliefs about the importance of exercise for osteoarthritis can influence my outcomes	Exercise
Exercise will cause my joint to wear out*	Exercise
Exercise will do more harm than good because I have osteoarthritis*	Exercise
Exercise will cause damage to my knee with osteoarthritis*	Exercise
My understanding of exercise for osteoarthritis can influence my outcome	Exercise for osteoarthritis
There are many things I can do to avoid flare-ups (of pain/swelling)	Flare-ups
Flare-ups (of pain/swelling) mean that I have damaged my joint*	Flare-ups
Understanding how my body protects itself can help me to avoid or reduce flare-ups (of pain/swelling)	Flare-ups
Inflammation is one way my body protects my joint from danger	Inflammation
My osteoarthritis involves too much body-wide inflammation	Inflammation
A flare in knee swelling means I have damaged my joint*	Inflammation
The amount of damage in my knee with osteoarthritis determines how much swelling I have*	Inflammation and damage
Other things happening in my body contribute to my knee osteoarthritis	Influence of variables
The people in my life have no influence on my pain*	Influence of variables
My surroundings have no influence on my pain*	Influence of variables
Things happening in my knee, the rest of my body and my environment can all change my pain	Influence of variables
My thoughts, beliefs, and ideas have no influence on my joint pain*	Influence of variables
My age determines whether or not I can improve my osteoarthritis*	Influence of variables
There are many things besides my joint that influence my osteoarthritis	Influence of variables
Learning about my osteoarthritis is an essential part of its treatment	Learning
I can help others with osteoarthritis by becoming more knowledgeable about it myself	Learning
My brain detects pain in my body*	Pain perception
My pain can be out of proportion to my osteoarthritis	Pain perception
My pain comes from my joints and travels to my brain*	Pain perception
The amount of damage in my knee determines the amount pain I feel*	Pain perception and damage
Physical activity/exercise is good for my osteoarthritis no matter how severe my osteoarthritis is	Physical activity/exercise
My osteoarthritis cannot be improved with physical activity/exercise*	Physical activity/exercise
Reflecting on my improvements and setbacks will help my osteoarthritis	Reflection
Thinking about how my osteoarthritis has changed will not influence my pain*	Reflection
The osteoarthritis in my joint can improve without surgery	Surgery
If my knee osteoarthritis becomes severe, I will need surgery*	Surgery

Items are sorted by the domain they relate to; future research will evaluate item order effects.

* Indicates reverse scoring

The Osteoarthritis Conceptualisation item bank uses a 5-point Likert-scale with responses ‘Strongly disagree’, ‘Agree’, ‘Neither agree nor disagree’, ‘Agree’, and ‘Strongly agree’ (these responses are displayed from left to right as written, but do not display numeric anchors–they are scored from 1 to 5 respectively). Twenty-three items require reverse scoring (Table 4). A higher score should reflect an understanding of osteoarthritis and physical activity that is more aligned with contemporary scientific understandings, with a range of possible scores between 45 and 225. Future research will evaluate the measurement properties of this questionnaire.

The expert panel reached consensus that the final item bank corresponded to the following domains: activity and damage; changeability, definition of osteoarthritis; physical activity/exercise (for osteoarthritis); flare-ups; inflammation (and damage); influence of variables; learning; pain perception (and damage); reflection; surgery (Table 4).

### Quantitative pretesting

#### Field testing

**Fig 3:**
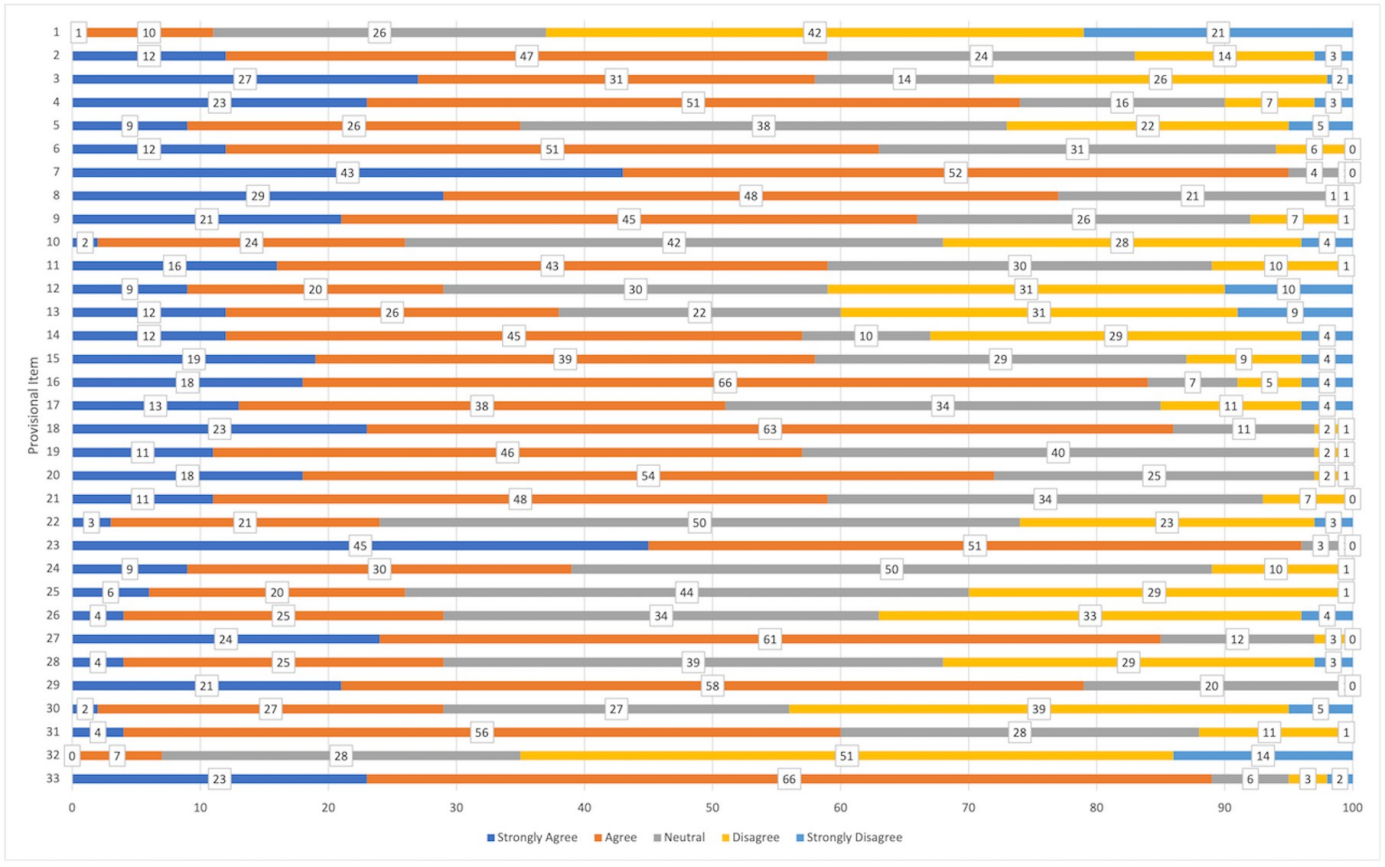
Response distribution for provisional item bank field testing.

Between August 2020 and December 2021, 100 people (68 female, 31 male) with painful knee osteoarthritis were recruited (Table 3). Seventy-nine people were recruited in South Australia and 21 were recruited in Victoria, Australia, with an average age of 66.9 (±9.0) years. Response distribution for each provisional item is reported in Fig 3. Overall response distribution is as follows: 3.4% ‘Strongly Disagree’; 15.9% ‘Disagree’; 25.9% ‘Neutral’; 40% ‘Agree’; 14.7% ‘Strongly Agree’. The online database used to collect data did not permit respondents to leave any questions blank, so there were no missing data in this dataset. The theoretical range of summed scores for the provisional item bank is 33 to 165. Field testing did not detect an overall floor or ceiling effect, with a mean and standard deviation for the summed score of 110.2 (±11.4), however, fifteen items were answered with the highest or lowest response choice by 15% or more of the population.

#### Survey quality prediction

Table 5 shows the characteristic differences between the provisional and final versions (see [75] for detailed characteristic definitions and coding instructions). The SQP evaluation highlighted that the provisional version requests that participants “please answer the question based on what you believe would be true”. This statement is not graded consistently with the response scale (i.e., the item response options use agreement/disagreement rather than true/false), and furthermore, it is unbalanced because it does not use the opposite sentiment statement (false) to avoid response bias. The final wording of the instructions addresses these issues.

**Table 5:** Comparison of characteristics identified by survey quality prediction.

Characteristic	Provisional Item Bank	Osteoarthritis Conceptualisation Item Bank
Domain	Health	Health
Domain: health	Personal physical health condition	Personal physical health condition
Concept	Complex concepts	Complex concepts
Concept: complex concept	Other	Other
Social Desirability	A bit	A bit
Centrality	Rather central	Rather central
Reference period	Present	Present
Formulation of the request for an answer: basic choice	Indirect request	Indirect request
WH word used in the request	Request without WH word	Request without WH word
Request for an answer type	Imperative	Declarative
Use of gradation	No gradation used	Gradation used
Balance of the request	Unbalanced	Balanced or not applicable
Presence of encouragement to answer	Encouragement present	Encouragement present
Emphasis on subjective opinion in request	Emphasis on opinion present	Emphasis on opinion present
Information about the opinion of other people	No information about opinions of others	No information about opinions of others
Use of stimulus or statement in the request	Stimulus or statement is present	No stimulus or statement
Absolute or comparative judgment	An absolute judgement	An absolute judgement
Response scale: basic choice	More than 2 categories scales	More than 2 categories scales
Number of categories	5	5
Labels of categories	Fully labelled	Fully labelled
Labels with short text or complete sentences	Short text	Short text
Order of the labels	First label positive	First label negative or not applicable
Correspondence between labels and numbers of the scale	Not applicable	Not applicable
Theoretical range of the concept bipolar/unipolar	Theoretically bipolar	Theoretically bipolar
Range of the used scale bipolar/unipolar	Bipolar	Bipolar
Symmetry of response scale	Symmetric	Symmetric
Neutral category	Present	Present
Number of fixed reference points	3	3
Don’t know option	DK option not present	DK option not present
Interviewer instruction	Absent	Absent
Respondent instruction	Present	Present
Extra information or definition	Present	Absent
Knowledge provided	Other explanations	—
Introduction available?	Available	Available
Request present in the introduction	Request not present	Request not present
Number of sentences in introduction	1	1
Number of words in introduction	12	13
Number of subordinated clauses in introduction	0	1
Number of sentences in the request	1	1
Number of words in request	25	19
Total number of nouns in request for an answer	3	2
Total number of abstract nouns in request for an answer	1	0
Total number of syllables in request	30	28
Number of subordinate clauses in request	1	1
Number of syllables in answer scale	10	13
Total number of nouns in answer scale	0	0
Total number of abstract nouns in answer scale	0	0
Showcard or other visual aids used	Not used	Not used
Computer assisted	Yes	Yes
Interviewer	No	No
Visual or oral presentation	Visual	Visual
Position	1	1

The provisional questionnaire had a predicted quality score of 0.471, reliability of 0.514, and validity of 0.916. The final item bank had a predicted quality score of 0.498, reliability of 0.547, and validity of 0.911.

#### Readability

Both versions of the item bank had similar readability statistics: the provisional item bank had a Flesch Reading Ease score of 57.7, the final item bank had a Flesch Reading Ease score of 55.2 (lower scores indicating greater difficulty), and both had a Flesch-Kincaid reading level of 8.2 (suggesting appropriateness for an eighth-grade reading level).

### Summary

Given the findings of quantitative pre-testing, no changes were made to the wording of the final item bank (Table 4).

## Discussion

We have developed an item bank using a mixed method, iterative process. The 45-item bank can now be further evaluated as a questionnaire to assess conceptual frameworks about osteoarthritis and physical activity for people with knee pain. The final item bank was developed by a panel of experts based on iterative application of both deductive and inductive methods, incorporating perspectives from people with painful knee osteoarthritis, quantitative field testing, and survey quality prediction assessment. The final item bank has been categorised into hypothesised domains for future factor analyses, has acceptable readability, and acceptable predicted validity and reliability.

Conceptual framework theory posits that many elements of cognition comprise a framework [23]. Proposing the development of a questionnaire that captures or assesses a conceptual framework assumes that conceptualisation is both the sum of many elements (e.g., knowledge, beliefs, experience), and that the framework becomes a single measurable construct in its own right. By considering how one integrates a wide variety of knowledge, belief, experience, and environmental stimuli when learning, it can be said that a conceptual framework is, to some extent, the sum of its parts. Using elements of conceptual framework theory that overlap with educational psychology [24], we have proposed a series of domains relevant to each item in this item bank, which all relate to conceptualisation about knee osteoarthritis and physical activity. These domains are similar to those identified by a wider group of people recovered from persistent pain, as being critical learnings to enable their recovery; [76] however it is important to acknowledge that these papers have overlap in authors. A recent survey aiming to evaluate osteoarthritis *knowledge* (using a true-false Likert scale) showed similarity to some of our generated items/domains [77], supporting external validity. That our instruments did not fully overlap also supports our evaluation of a more complex construct of conceptualisation of which knowledge is one aspect. Regardless, independent replication of these results is warranted.

Survey quality prediction can offer guidance for the development and modification of individual characteristics of assessment tools. Predictions for reliability and validity should specifically not be interpreted in the same way as empirical evaluations of these constructs, because of the fundamentally different methods by which they are derived. Perhaps the most impactful use of survey quality prediction is the consideration of detailed questionnaire characteristics, rather than interpretation of predicted values that cannot replace real-world evaluation of scale properties. In the process of developing this item bank, important changes were made to the instructions of this questionnaire. Based on the qualitative pre-testing and expert appraisal, we are confident that these changes were important and necessary, but the small change in predicted quality/reliability/validity may suggest that these changes did not overly worsen or improve the quality of the questionnaire in context of the SQP 2.1 algorithm [68]. Regardless, evaluation of multiple domains of reliability and validity via prospective studies are essential to further develop this questionnaire.

The skewed distribution of responses favouring agreement over disagreement in the provisional item bank raises the possibility of a sampling bias. Field testing was conducted in a cohort who had enrolled in an intervention study, where they were informed that they would receive education about osteoarthritis and pain, and a physical activity program for knee osteoarthritis. This cohort may have already been more agreeable to concepts such as ‘physical activity is important for osteoarthritis management’, than the wider osteoarthritis-affected population. No floor or ceiling effects were present in the overall summed score for the provisional item bank [66, 67], but further evaluation of skewed items, and expanding the sample beyond an intervention study, would seem warranted.

The panel considered the possibility that respondents may find a forced choice item (i.e. no ‘unsure’ option) frustrating, and thereby reduce completion rates [78]. However, the panel voted in favour of changing the ‘Neutral’ response option to ‘Neither agree nor disagree’ for two reasons: the utility of the tool would be compromised if too many participants selected the ‘unsure’ response [79], and that including an ‘unsure’ response option would suggest to participants that this questionnaire is an assessment of knowledge, rather than of agreement with the item.

Both the provisional and final versions of the item bank scored appropriately at an eighth-grade reading level. Previous work has classified Flesch Reading Ease scores of 50 and 66 as ‘fairly difficult’ and ‘standard’ respectively [80]. Importantly, there is a paucity of data on health literacy and older adults [81]. However, both the Australian Bureau of Statistics [82] and the Centers for Disease Control [83] have reported that health literacy is generally low among older adults, and the Centers for Disease Control recommends health materials be presented as simply as possible to maximise the usefulness of these materials [83]. It will therefore be critical to consider item reading ease during the item reduction phase of scale development (i.e., consider that item function may be impacted by readability).

We used a pragmatic combination of inductive and deductive guideline recommended methods with an iterative and reflective approach. While scale development guidelines offer recommendations for highly rigorous development of valid and reliable outcome measures [36, 45, 60, 65, 79, 84], tools for health research are often criticised for poor or incomplete development [45, 85]. We raise the possibility that not meeting development guidelines may be due to pragmatic reasons. For instance, scale development guidelines typically suggest undertaking separate deductive and inductive pathways for item development, all in separate studies [36]. With the literature of psychometric health assessments, this recommendation is largely adhered to: 83% of studies used solely deductive item development methods; 11% used solely inductive methods; and only 6% used a combination of both methods [36, 45, 60, 65, 79, 84]. However, completion of separate deductive/inductive lines of research may not be possible in situations of restricted funding and/or limited research infrastructure.

A combined inductive and deductive approach may provide benefit when a construct is novel, such as conceptualisation of osteoarthritis pain/activity. The preliminary items derived deductively by the expert panel had low agreement for domain specificity (by that same expert panel), suggesting that deductive development pathways have limitations. Our use of inductive procedures via qualitative cognitive interviewing refined the items and successfully facilitated re-classifying to relevant domains. These findings support the ability of empirically driven development to improve content validity and contribute to the theoretical process of a conceptual framework that may have been missed, had we only used deductive methods [86]. Indeed, past work discussing the development of new measures for osteoarthritis has raised this idea, that iterative and reflective approaches integrating elements from different scale development methods underpinned by pragmatic decision-making may facilitate more theoretically and empirically valid scale development [86].

### Strengths and limitations

This study’s primary strength is the integration of guidelines to mitigate many of the problems associated with health assessment tool development. Doing so has challenges, namely extending the duration and cost of development from initial planning to final clinical application. However, this process brings advantages of rigour, transparency, and content validity. The use of a combination of inductive and deductive methodology facilitated the integration of expert and end-user feedback. Members of this panel have previously experienced rapid scale development without the use of guidelines, and this has a demonstrably negative impact on the validity of the resultant tool [87]. Here, we lodged and locked the study protocol as is recommended [38]. We have also used a combination of expert and consumer insight for estimating content validity of the item bank prior to further scale development. The use of a combination of qualitative and quantitative methods provides comprehensive scale evaluation. Finally, we adhered to guidelines for method and analysis reporting [47, 48]. This study also has limitations, most notably the limited representation of the recruited consumer and expert populations. While the expert panel had representation across research, health education, and clinical practice, the experts only included those with pain expertise and allied health backgrounds. All participants were recruited in Australia, and most consumer participants in both the cognitive interviewing and field-testing groups had completed high school and some tertiary education. While this population may not be representative of the target population in Australia, we purposively sampled from both the community and a group of people who had participated in pain science education, to incorporate a variety of conceptualised experiences. Future testing should evaluate face validity and comprehension of the item bank in other populations, especially those with lower educational levels. While a wide scope of qualitative work was considered in the development of this questionnaire, this was an informal approach–a systematic review was not undertaken. This raises the possibility that relevant information from existing literature was missed. However, the cognitive interview process increases our confidence that the questionnaire contains items that are meaningful to people with lived experience. Lastly, all cognitive interview data was collected and presented to the expert panel via one researcher (BWP)–this introduces a potential bias regarding what data were put forward for review.

In summary, we have completed the first step in developing the Osteoarthritis Conceptualisation Questionnaire, by comprehensively generating items for evaluation, pretesting those items, and identifying domains of interest. Future work is warranted to evaluate the psychometric properties of the item bank in a large-scale sample, which will assist in the refinement of this final item bank into a research and clinical tool that allows us to better capture how people with osteoarthritis conceptualise their osteoarthritis and the role of physical activity in its management.

## Supporting information

S1 Appendix Cognitive interview script. (DOCX)

S2 Appendix Cognitive interview script trial participants. (DOCX)

S3 Appendix Provisional questionnaire. (DOCX)

S4 Appendix Quotation synthesis pre-appraisal. (DOCX)
