# Supplementary material for: Item development and pre-testing of an Osteoarthritis Conceptualisation Questionnaire to assess knowledge and beliefs in people with knee pain
Source: PLoS One. 2023 Sep 29;18(9):e0286114. doi: 10.1371/journal.pone.0286114 (PMC10540977; doi:10.1371/journal.pone.0286114)
Supplement: S2 Appendix — (DOCX) [file pone.0286114.s002.docx]

**S2 Appendix. Cognitive Interview Script Trial Participants**

**Interview 1**

[This interview will be conducted in writing or online]

The purpose of this interview is to learn about how you understand this survey before it is used for larger research studies. We aren’t concerned about your responses to the actual questions themselves. It’s important to get your feedback on these questions, so that our future research can be as effective as possible.

First, I’d like to start by asking you about a few key phrases. If you don’t recognise a word or phrase, that’s ok, this is important information for our research.

What does [term] mean to you? Ok, what does [term] mean to you? [*Repeat for each term*]

[Terms: osteoarthritis, outcome, pain, inflammation, exercise, physical activity, flare-up, activity, protective system]

Now I’d like you to tell me, out loud, everything that passes through your head while answering each question, and to do so without planning what to say while you’re thinking.

Let’s start with an example before we begin. Try to visualise the place where you live, and think about how many windows there are in that place. As you count up the windows, tell me what you are thinking about. [Adapted from (Willis 1999)]

Again, the purpose of this interview is to learn about how you understand these questions, so there are no right or wrong answers, and we aren’t concerned about your answers to each question on the paper. Do you have any questions?

[The remainder of Part 2 should be completed for each item before continuing to the next item. Complete the “think aloud”, questions about item difficulty, and questions about item meaning before moving on to the next item]

Great, now we’re going to go through the survey. One at a time, **read each question aloud, and tell me everything that passes through your head while answering** each question.

[If participants **do not** mention any problems or difficulties with the question (understanding, comprehension, interpretation, etc.), use the following questions:]

- - **Is anything about that question challenging?**
  - **Were there any words or phrases that you did not understand or that were unclear to you?**

**Can you tell me in your own words what that question was asking?** (Beatty & Willis 2007)

**Interview 2 - Part 2**

Now that we’ve gotten through each individual question, I’d like to ask you some questions about the survey as a whole.

What do you think the questionnaire is about? If you are unsure what it is about, what is your best guess?

How did you feel when you filled out this questionnaire?

Do you think this questionnaire asks important questions about your circumstance?

If so: describe how those questions are important to you.

If not: why do you think the questions are not important?

Can you relate to the questions in this questionnaire? If yes, in what way?

“Were there any questions/statements that you originally did not understand when you completed the questionnaire, but that now make more sense after participating in the treatment?”

Our goal with this questionnaire is to ask questions about how people understand and think about their knee osteoarthritis. With that in mind…

Are there additional questions you think this questionnaire should include? If yes, which one(s) and why?

Tell me a bit more about content you think should be included in this questionnaire?

Are there any questions you think are irrelevant to you and should be removed? If yes, which one(s) and why?

Do you have any other comments/thoughts about this questionnaire?

Finally, when reflecting on the treatment you received as a whole:

“What was the most important thing you learned from the treatment?”

“How did learning this impact your knee and your life more generally?”
