# Supplementary material for: Item development and pre-testing of an Osteoarthritis Conceptualisation Questionnaire to assess knowledge and beliefs in people with knee pain
Source: PLoS One. 2023 Sep 29;18(9):e0286114. doi: 10.1371/journal.pone.0286114 (PMC10540977; doi:10.1371/journal.pone.0286114)
Supplement: S3 Appendix — (DOCX) [file pone.0286114.s003.docx]

Appendix 3: Provisional Questionnaire

**NOTE: THIS IS NOT THE FINAL ITEM BANK**

1. The outcome for my knee is set in stone

2. There are many things in the rest of my body that contribute to my knee osteoarthritis

3. The place I’m in and the people I’m with have no influence on my pain

4. The amount of pain I have relates to the amount of damage I have in my knee

5. My pain emerges from my brain in response to things happening in my knee, the rest of my body and the world

6. Osteoarthritis is changeable without surgery

7. Learning about my osteoarthritis is an essential part of its treatment

8. Physical activity/exercise is good for osteoarthritis no matter how severe the osteoarthritis is

9. Inflammation is one way the body protects the joint from danger

10. I can sometimes overprotect my joint by making too much pain

11. My pain comes from my joints and travels to my brain

12. My osteoarthritis will get worse over time no matter what I do

13. My thoughts, beliefs, and ideas do not influence my joint pain

14. My age does not determine whether or not I can improve my osteoarthritis

15. “Bone-on-bone” is an accurate descriptor of my osteoarthritis

16. “Wear and tear” is an accurate descriptor of my osteoarthritis

17. I can help others with osteoarthritis by becoming an expert on it myself

18. Reflection on my progress, no matter how small, will help my osteoarthritis

19. Because osteoarthritis is more than my knee, I can create my own future for my knee

20. Because osteoarthritis is more than my joint, there are many different ways I can improve my symptoms

21. Exercise can reduce joint inflammation

22. When osteoarthritis becomes ‘bone-on-bone’ physical activity can no longer help; only surgery can help

23. Knowledge about osteoarthritis can help me to exercise in a safe way

24. Exercise can make the cartilage in my knee healthier

25. Doing more activity than normal can further damage my joint

26. Osteoarthritis must be rested

27. Slowly increasing my activity level over time increases how strong my body is, which allows me to do more activity than I previously could, without doing damage to my joint

28. Exercising contributes to the ‘wear and tear’ of my osteoarthritis

29. My knowledge and beliefs about osteoarthritis and about exercise can influence how well I do

30. There is little I can do to avoid flare-ups (of pain/swelling)

31. Flare-ups (of pain/swelling) are warning signs that I have injured or damaged my joint

32. My osteoarthritis cannot be improved with physical activity/exercise

33. Having an increased understanding of my body’s protective systems can help me to avoid or reduce flare-ups
