# Supplementary material for: Item development and pre-testing of an Osteoarthritis Conceptualisation Questionnaire to assess knowledge and beliefs in people with knee pain
Source: PLoS One. 2023 Sep 29;18(9):e0286114. doi: 10.1371/journal.pone.0286114 (PMC10540977; doi:10.1371/journal.pone.0286114)
Supplement: S4 Appendix — (DOCX) [file pone.0286114.s004.docx]

**S4 Appendix. Quotation Synthesis Pre-Appraisal**

OACS Development Meeting Agenda

*Expert Appraisal of Cognitive Interviews for Content/Face Validity*

Date: 2.9.2021

Present: BWP, TRS, FAB, DB, LM, MC

Notes:

A brief presentation outlining the methodological process for the meeting will precede the review of the following interview content.

[Phrases in brackets came from written feedback prior to the cognitive interview]

(Phrases in parentheses are asides or paraphrasing)

**Summary of participants:**

N=18 (10 female, 8 male)

n=15 recruited from trial ineligibility list and community

n=3 recruited from EPIPHAKNEE trial pilot sample, having completed the intervention

Age M(SD): 71.8 (7.5)

Education level:

1: did not complete high school

5: completed high school

3: enrolled or completed a non-university qualification

4: enrolled or completed a university qualification

5: enrolled or completed a university post-graduate degree

1. The outcome for my knee is set in stone.

Alt: remove period

001: n/a

002: n/a

003: n/a

004: n/a

005: n/a

006: n/a

007: n/a

008: n/a

009: n/a

010: n/a

011: n/a

012: n/a

013: n/a

014: n/a

015: n/a

016: n/a

017: n/a

018: If you didn’t understand that (set in stone) meant, it could be quite difficult to answer.

1. There are many things in the rest of my body that contribute to my knee osteoarthritis

Notes: Participants report neutral or unsure option being important for fact-based item

001: n/a

002: n/a

003: n/a

004: n/a

005: n/a

006: I've disagreed with that because I just don't have any arthritis of any kind, anywhere else in my body.

007: I find a little bit obscure and a little bit confusing…. perhaps having the unsure might be a good thing.

008: n/a

009: n/a

010: n/a

011: n/a

012: the word osteoarthritis

013: n/a

014: n/a

015: n/a

016: n/a

017: n/a

018: n/a

1. The place I’m in and the people I’m with have no influence on my pain

Alt: My environment and the people around me have no influence on my pain

001: “I wasn't sure if that was a **psychological place or a physical place** as in a home.”

002: n/a

003: n/a

004: no, nobody has any influence on the pain. Sort of can't understand any more of that. I don't know whether it relates to, if I was working, mixing with people with pain or I don't know. I don't, no, I can’t understand that one. I really don't know how other people can influence your pain. And I don't know, um, the place that I was in, I don't know how that could influence my pain

005: n/a

006: n/a

007: I don't know whether these, this one is particularly relevant, um, at all. I'm in many places, so I don't know that that, um, is relevant to a particular answer and the people I'm with, with lots of people and doing lots of things and working and not working. I'm not confused about the words, obviously I understand the words. I just don't know how to respond to this particular question.

008: n/a

009: I'm not quite sure what. What you're after in that question. If you, if you're in a very rocky place or something it'd have an effect, but yeah, no, I can't, I can't see the relevance of the question really?

010: n/a

011: n/a

012: n/a

013: n/a

014: n/a (unsure option: “I think that would be worthwhile”)

015: ["Place I'm in" could be interpreted as either physical location or (I presume) emotional /wellbeing state.]

016: n/a

017: the place on me might be a physical place or in your head place

018: n/a

1. The amount of pain I have relates to the amount of damage I have in my knee

Notes: Participants report neutral or unsure option being important for fact-based item

001: n/a

002: n/a

003: I honestly can't answer that one. Okay. Because I have so much damage on my legs. The knee part is irrelevant to the rest of it. Okay. Okay. So I'd say neutral.

004: n/a

005: n/a

006: n/a

007: I guess I possibly don't know enough about my osteoarthritis to, to give that a particular agree or disagree because this is the first time that I've experienced any of this.

008: n/a

009: n/a

010: n/a

011: n/a

012: n/a

013: n/a

014: n/a

015: n/a

016: I don’t agree with it, but it’s very clear

017: n/a

018: You could answer (agree) or…disagree with that because there were other factors that related to it so that one could be slightly unclear.

1. My pain emerges from my brain in response to things happening in my knee, the rest of my body and the world

Alt: My pain emerges from my brain in response to things happening in my knee, the rest of my body, and my environment

001: n/a

002: I'm really not quite sure where the pain is and why you mentioned the pain comes from my brain

003: n/a

004: if you have pain, your brain tells you that you're having pain. I think its vice versa, I don't know, nothing, from nothing from the world would make any difference there.

005: **“The entire sentence is confusing - are you asking me to respond based on agreeing/disagreeing with all the factors?”**

006: “**I’m not sure about ‘the rest of my body and the world’**… someone yells at me and my knee hurts more… that doesn't make sense.

007: obviously I believe that, that, you know, the pain that I experienced is real because I have and I don't necessarily agree, um, with that has anything to do with the world out there it's, obviously my body's response to what's happening. I understand the general question I just don't, uh, don't necessarily agree with the last bit of it. ‘The rest of my body and the world’ I'm finding that a bit, a bit strange. I don't know whether this particular question Brian is a really important to ask.

008: [AII of it.] **it just seemed like a, yeah, like a cosmic question**, I guess. It was probably the last bit, like I said, the rest of my body and the world that cosmic bit that threw me. Why would, why would the world have any bearing on whether my knee hurts or not?

009: n/a

010: n/a

011: **the world seems to say it’s psychosomatic** and I won't even consider that for a moment

012: n/a

013: n/a

014: n/a

015: [How do I answer if I think that most of my knee pain comes from the joint, or connected muscles/ tendons ie from my body BUT there are times when my pain threshold is higher from my body's reactions to external matters. There is no 'sometimes/ occasionally' option to select.] It may do, but that’s not how I perceive it.

016: Reasonably clear, but I suspect it will be interpreted in different ways, because pain itself doesn’t emerge from the brain. It emerges in response to signals that travel around.

017: **I don’t know about that word ‘emerges’**. **‘And the world’?, I don't know about that**

018: **You could add something to the end like …the world events**.

1. Osteoarthritis is changeable without surgery

001: n/a

002: n/a

003: n/a

004: n/a

005: “My knee could be treated effectively without me necessarily learning about it”

006: n/a

007: n/a

008: n/a

009: n/a

010: n/a

011: n/a

012: n/a

013: n/a

014: n/a

015: ["changeable" - what do you mean? impacts of o/a can often be reduced or managed but not remove o/a]

016: n/a

017: I don’t know about changeable, but it's certainly manageable without surgery

018: n/a

1. Learning about my osteoarthritis is an essential part of its treatment

001: n/a

002: n/a

003: n/a

004: n/a

005: The definition of essential could mean different things to different people

006: n/a

007: n/a

008: n/a

009: n/a

010: n/a

011: n/a

012: n/a

013: n/a

014: n/a

015: n/a

016: n/a

017: n/a

018: n/a

1. Physical activity/exercise is good for osteoarthritis no matter how severe the osteoarthritis is

Alt: Physical activity/exercise is good for severe osteoarthritis

001: n/a

002: n/a

003: n/a

004: n/a

005: “Um, I understand what the question is asking, but maybe, uh, people willing to prep severe in different ways. Um, and if you take that to the absolute limit, um, when it's very severe, um, people might, if they use that as a basis, **they might answer, um, disagree, even though they know that physical activity is good**….I think it’s the ‘no matter’.”

006: n/a

007: n/a

008: n/a

009: n/a

010: n/a

011: n/a

012: n/a

013: n/a

014: n/a

015: n/a

016: n/a

017: It’s always good. **I mean you don’t have to go too strong**.

018: n/a

1. Inflammation is one way the body protects the joint from danger

Notes: Participants report neutral or unsure option being important for fact-based item

001: n/a

002: perhaps I'd say inflammation is the way the body, um, warns you, that things aren't right

003: It's not the inflammation. Isn't the one that protects the joint from danger. It's the pain, not the inflammation. How does an ordinary person who has no medical note understand inflammation, but they can understand pain.

004: n/a

005: I thought, well, I don't know the answer, so why don't **I put neutral because I don't know the answer.** The question is clear, but I just didn’t know the answer

006: I don’t know enough to comment. **[Participant reported that an ‘unsure’ option would be helpful here]**

007: most people would, would have to understand a lot about inflammation to, to actually answer that one or another.

008: n/a

009: **Maybe I’m not quite sure what inflammation is**. I thought it was, you know, within my thumbs, they get inflamed, they sort of fluid sort of builds up in them

010: **{What is this question asking?} if you understand what inflammation is**

011: n/a

012: n/a

013: n/a

014: **I don’t know enough about that to comment**

015: n/a

016: n/a

017: n/a

018: n/a

1. I can sometimes overprotect my joint by making too much pain.

Alt: removed period

Alt: Sometimes pain causes me to overprotect my joint

001: “I would have preferred by anticipating too much pain. Anticipating means that, you know, it's going to hurt. Making. I don't know how you make pain. You can anticipate it coming … but I don't know how you can make pain for yourself.”

002: “ ‘I can sometimes overprotect my joint’, but not add ‘by making too much pain’.” … “How can you make too much pain?”

003: I don't understand the question…I do overprotect my knee. nobody in the right mind would do things to make the pain worse. Okay... Yeah, that might sound tough, but I protect myself as a fault. I don't have extra pain.

004: n/a

005: I really am stumped. I'm not sure what it's asking. Unless it was saying when I'm in too much pain, I overprotect my joints. So if I happen to be in a lot of pain, I will take steps deliberately not to use it or I'll make sure I rest it. [Suggested change]: When I'm in too much pain, I can sometimes overprotect my joint.

006: Does that imply that when I feel more pain than normal, I shut down more than I should? Just a bit unclear about that one. What, what does it mean by making too much pain? Does that mean causing doing something that causes the pain or is it implying? Feeling the pain?

007: I don't know what they mean by saying, making too much pain.

008: [The whole statement does not make sense.] I do say to people in conversations when my knees hurting and on carry on walking and I say to it that I'm hurting it back. That's my way of carrying on. [Later, after reflecting on original answer:] That doesn't sound right. As in, how can you overprotect it if you're making it hurt more?

009: [making too much pain] that doesn't really make sense to me because if it was, you making too much pain, you'd stop. I think the whole thing is a bit unclear

010: what's challenging about it is some people aren't prepared to take maybe a risk or there's a risk, or they can't, they don't want pain, they can't handle pain. So therefore they just don't do it. They tell themself don't do it cause you're going to hurt yourself.

011: I don't understand. Is it all actually on secret? Okay. I would say I can, sometimes I protect my joint from making too much pain.

012: **I would not normally make pain**, you know, not normally create pain… for the sake of it

013: **by making too much pain, I wouldn’t go down that track**.

014: **What does it mean by making too much pain**? Does that mean doing something that causes the pain or, or is it implying feeling the pain?

015: [I'm not clear about the meaning of this - infers that I (my mind) create the pain to protect the joint - is this the intent of the question?] It appears that it's saying that mentally or creating pain to protect the joint. I don’t really agree with that.

016: This was not a clear statement. Um, I mean, grammatically making too much pain, doesn't make a lot of sense. Um, I mean, **I understand I can sometimes overprotect my joint**, which means that, um, we shy away from doing anything at all that we think might cause an issue or might cause pain in some ways. So that it's fine. Um, the phrase by making too much pain, I mean, you could use the word faking instead of make, but to me that, that terminology is not right. [What would be a better alternative do you think?] I would say by, well, even just by saying, **by making too much of my pain.**

017: How do you overprotect a joint by making too much pain? I don’t’ get that. **Maybe it's saying that if you do a whole lot of something that causes a lot of pain after that, you won't do anything for a while to make up for that.**

018: If somebody didn't know overprotect, that word could be a bit challenging. **If you'd done the study, you would know what it meant**, but if you haven't done the study, you’d be going, ‘Over protect what the heck?’ How can I overprotect by pain?

1. My pain comes from my joints and travels to my brain

Notes: Participants report neutral or unsure option being important for fact-based item

001: n/a

002: n/a

003: n/a

004: n/a

005: As above - **I don't know the answer so not sure if I am supposed to guess or answer neither**

006: n/a

007: Obviously to me, this means that, you know, any time you have pain in any parts of your body, the brain will stimulate. Uh, a, a result and, uh, yes, that did that. That's when you feel pain because the brain was **send messages to the rest of your body to say that you are in some sort of discomfort**.

008: That's the same as question five

009: n/a

010: n/a

011: n/a

012: n/a

013: n/a

014: n/a

015: n/a

016: n/a

017: Maybe you could say “**A message comes from my joints and is sent to my brain**”

018: n/a

1. My osteoarthritis will get worse over time no matter what I do

001: n/a

002: n/a

003: n/a

004: n/a

005: n/a

006: n/a

007: n/a

008: n/a

009: n/a

010: n/a

011: n/a

012: n/a

013: n/a

014: n/a

015: n/a

016: n/a

017: n/a

018: could be slightly unclear but I understand it.

1. My thoughts, beliefs, and ideas do not influence my joint pain

Alt: My thoughts, beliefs, and ideas **do not** influence my joint pain

001: The pain concept is thoughts, beliefs, and ideas I've just lumped those all. I actually lumped all those together as anticipation of pain. Had it been worded, ‘my thoughts, beliefs and ideas influence my joint pain.’ I would have said that they can, but in my case, I've tried to override that so that it doesn't influence moving the joint. So **I'm not sure about the negative ‘do not’**

002: BRIAN: “Can you reread that statement” 002: “…I took it as, um, ideas do influence my joint pain, but yet do not. **Yes. Now I disagree with that**. **It's tricky**.”

003: I **disagree**. My thoughts **don't** influence my pain.

004: n/a

005: I would say I disagree that they do not influence my joint pain.

006: [Agree] I don't think that that there's anything very significant in my, um, self-awareness that influences how painful it feels.

007: **I'm just not sure how to respond to that one at all**.

008: I reckon I would have said agree. Um, now rereading it because yeah. You can have positive thoughts and try and block the pain out. [mumbles rereading question] **no sorry. I'm getting lost**. Yeah. My thoughts, beliefs ideas. Didn't influence my joint. Yes, so. I would have said disagree.

009: n/a

010: It doesn't influence me

011: n/a

012: n/a

013: n/a

014: n/a

015: n/a

016: ideas, it's such a widespread concept. Um, I mean, I'd prefer to use a word like knowledge. Because I think knowledge is more concrete

017: n/a

018: n/a

1. My age does not determine whether or not I can improve my osteoarthritis

Alt: My age **does not** determine whether I can improve my osteoarthritis

001: **I wonder if that would have been better worded ‘my age determines whether or not I can improve my osteoarthritis.’** Um, **by putting the negative in there, um, colours, how that question is answered.**

002: **you put not in there. And then that changes how I should answer**

003: It's not going to get better. Okay. So it's not going to improve my age does not determine. So my age does not determine, I agree with that. It's my age. That does not determine whether my…

004: n/a

005: n/a

006: n/a

007: does determine wear and tear obviously in your body, but then I agree that you can do whatever regardless of age to try and improve your situation.

008: **means age has got nothing to do with it. Yeah, I don't reckon it does**. You could have osteoarthritis when you're younger

009: n/a

010: n/a

011: n/a

012: n/a

013: n/a

014: n/a

015: n/a

016: n/a

017: n/a

018: n/a

1. “Bone-on-bone” is an accurate descriptor of my osteoarthritis

001: n/a

002: n/a

003: n/a

004: n/a

005: n/a

006: n/a

007: n/a

008: n/a

009: n/a

010: n/a

011: n/a

012: n/a

013: n/a

014: n/a

015: n/a

016: n/a

017: n/a

018: n/a

1. “Wear-and-tear” is an accurate descriptor of my osteoarthritis.

Alt: removed period

001: n/a

002: n/a

003: n/a

004: n/a

005: n/a

006: n/a

007: n/a

008: n/a

009: n/a

010: n/a

011: n/a

012: n/a

013: n/a

014: n/a

015: n/a

016: n/a

017: n/a

018: n/a

1. I can help others with osteoarthritis by becoming an expert on it myself

Alt: I can help others with osteoarthritis by becoming more knowledgeable about it myself

001: **Becoming an expert would probably better if it was becoming more knowledgeable**, because when you say expert, you think that somebody that's done years and years and years of study, and know as much as there is to know about a joint. So. I would say I'd like to be as knowledgeable as possible without being, you know, the chief surgeon or anything like that.

002: **just leave out the expert**, word for me

003: n/a

004: n/a

005: it was **the word expert that I found confusing in here**. Um, to me, **if you just said, um, becoming more knowledgeable**, uh, that would make me more comfortable with answering

006: “**Not sure about being an expert but clearly the more knowledge** I've got, um, then even in casual conversations with others, I can be more supportive and perhaps even help each other. [Marked neither, “It sort of makes sense, but I’m not sure if it’s entirely true…I was a bit doubtful that I'd ever be able to say that I'm an expert on it myself”]

007: Okay. I don't know that I've ever become an expert. I find perhaps the wording as expert, not, **I don't like the word expert in there.** Expert is not a necessary word in there, perhaps, perhaps something like, you know, uh, researching, being more well-informed about your own situation might be more appropriate.

008: You can help others. By um, passing on information. **I don’t know if expert is the right word**. Just by learning I suppose, innit?

009: Uh, no, I, I don't think, uh, really, you'd need to be a doctor just as a general person. I don't, I don't think I could really help other people.

010: n/a

011: n/a

012: n/a

013: I don't think expert would be, **don't think Australians really respond to experts in the private world. I think they call it smart ass**

014: **Not sure about being an expert**, **but clearly the more knowledge** I’ve got, um, then even in casual conversations with others, I can be more supportive…

015: n/a

016: n/a

017: n/a

018: **Maybe the word expert might be better with that becoming as knowledgeable** as I can on it myself.

1. Reflection on my progress, no matter how small, will help my osteoarthritis

Alt: Reflection on improvements, even small improvements, will help my osteoarthritis

001: n/a

002: n/a

003: n/a

004: n/a

005: I thought, **well, what if there is no** **progress**? I would agree in some sense, but I might disagree in other senses dependent on the circumstances. I thought the question was fairly straight forward, but it just did, um, conjure up more questions.

006: n/a

007: Well, I don't know. **I don't know whether it's just the reflection word that just, um, may, may be a little bit of a concern for some people.**

008: how do I know I'm progressing? Like it can hurt one day and you know, another day it's is fine or even during the day, unless I'm, unless maybe I'm visiting a physiotherapist or a doctor treatment, and they are telling you that you're **progressing**

009: [**I am not sure what is meant by reflection of my progress**] {What do you think is meant by the phrase reflection on my progress} um, as you think of it, think about what, what differences being made, uh, yeah. That, that, that could help in some small way sort of thing. Thinking about the any progress you are making and would help. Well, I'm just saying that, uh, no matter how small, uh, that would help a little bit anyway [**Participant indicated that the amount of impact of reflection on progress would be small**]

010: n/a

011: n/a

012: n/a

013: n/a

014: n/a

015: n/a

016: n/a

017: n/a

018: n/a

1. Because osteoarthritis is more than my knee, I can create my own future for my knee

Alt: Because osteoarthritis is more than my knee, I can change the outcome for my knee

001: **those last three words, as I said, I think I'd probably have left those out** because you can create your own future by doing different activities at different levels.

002: n/a

003: n/a

004: n/a

005: Um, osteoarthritis is more than my knee. I'm not sure what that is you saying? Um, I don't know what it would be distinct. Um, and then the second part seems I can create my own future from my knee. Uh, that seems to be a standalone statement to me. [Participant suggests changing to]: “I can influence my own future for my knee”.

006: ‘**osteoarthritis is more than my knee’… in my case, in my body, it's not**, but I'm conscious in the big wide world, big picture, it is.

007: I sort of understand the wording in some sense, but **I find this one confusing** and I think other people may, may agree with this that uh,, you know, this one is, it is definitely an unsure for me. I'm just not wrapped in the way that that's written.

I think sometimes Brian it's just, it's just the wording. I just, I'm just finding that a little bit difficult to get my head around. And I don't know whether others would feel exactly the same I can't obviously speak for others, but this is, this is the whole point of this that, um, you know, perhaps it needs to be a little bit more basic in what it's answering in that one.

I think, you know, throwing things in, like, I can create my own future for my knee and osteo arthritis is more than my knee people will go. Oh, I'm not even sure what that means.

[Participants suggested rewording: “I can create an outcome for my own knee from trying to find out more about what's going on with me.”]

008: [**Sounds a bit too cosmic**!] you can read stuff about power of thought, you think, aw Jesus, you know, that's, that's a bunch of old hippies or yeah.

009: [I have severe osteoarthritis in my lower back. This will always influence my knee]

010: n/a

011: n/a

012: n/a

013: n/a

014: in my body's case, it's only, it's only my knee and I'm not sure about. Um, recognizing that it's a bigger picture issue is going to create a different future for me.

015: I agree with that, but I'm a bit, I find the wording a bit awkward. I guess it just not words I would use

016: n/a : I can create my own future for my knee. **I mean, what does that mean?** I understand that to mean a **positive outcome for my knee**, but I’m not sure that everybody would see it that way.

017: **This last bit, I can create my own future for my knee, it sounds a bit, I don’t know, over the top**. I can create my own future for my whole body, not just the knee.

018: When you say create my own future, it's maybe for me has slight connotation of, you know, well, you know, like the more positive I am, I can keep curing this

1. Because osteoarthritis is more than my joint, there are many different ways I can improve my symptoms

001: n/a

002: n/a

003: n/a

004: n/a

005: (Participant suggested change) Because osteoarthritis affects more than just the joint…

006: because it's only in that one joint, I'm not sure that that's true, but I’m conscious there are probably different ways I can improve.

007: …and here we go again with ‘is more than my joints’ that I just, you know, for me, the osteo is, I wouldn't even put in about more than my joint.

008: n/a

009: n/a

010: n/a

011: n/a

012: n/a

013: n/a

014: n/a

015: n/a

016: n/a

017: n/a

018: n/a

1. Exercise can reduce joint inflammation

Alt: Moderate exercise can reduce joint inflammation

Notes: Participants report neutral or unsure option being important for fact-based item

001: **mild exercise** or **graded exercise** can help with that and help the body clear that inflammation.

002: n/a

003: n/a

004: n/a

005: I don't know the answer to this one. I think the question is straightforward, but again, it would be one of those questions where I would, if I had a choice as say, um, don't know, unsure.

006: **I'm a bit neutral on that because I feel as if I don't know enough**, but I'm also a bit cynical because my own experience is that, um, exercise is one of the things that does make it worse. I'm not sure I know enough. I'm not aware of any **particular exercise** I could do that would reduce the symptoms.

007: I probably need to, for my own personal situation, look at that a little bit more and see whether the **constant exercising** and there's all sorts of **different exercises** that actually assist with that inflammation

008: **I would have said unsure** because until you get medical advice or you read something that tells you that exercise can reduce joint inflammation, I would have said no because inflammation is like, the fluid innit that usually fluid and it tightens everything up

009: How, **how vigorous the exercise**?

010: n/a

011: n/a

012: n/a

013: Well, **I think mild exercise, probably not, not strenuous exercise**

014: **I'm not sure I know enough**.

015: **I don’t really know if that’s the case medically**

016: I have a problem with that statement. Um, not because it's not clear, but because I think that, um, **it's almost like putting fuel on the fire as well**. Um, I mean, th there are certain kinds of exercise that could be helpful. Um, but if I look at that statement just on the time, um, I could interpret that as being, um, I've got a swollen me, therefore I need to walk more. **you could use a word like different exercises. Okay. And that may be enough.**

017: n/a

018: n/a

1. When osteoarthritis becomes “bone on bone” physical activity can no longer help; only surgery can help.

Alt: removed period

Notes: Participants report neutral or unsure option being important for fact-based item

001: n/a

002: It depends what they call bone on bone

003: n/a

004: n/a

005: n/a

006: again, **I'm feeling neutral on that because I just don't know enough**

007: Um, it depends on how much that they are, uh, damaging their knees and I just believe that physical activity is, is the key to helping with everything.

008: I do agree with that. So that one's pretty straightforward I reckon.

009: n/a

010: n/a

011: n/a

012: n/a

013: n/a

014: again, it's a little bit about, um, having more knowledge, isn't it?

015: n/a

016: n/a

017: n/a

018: n/a

1. Knowledge about osteoarthritis can help me to exercise in a safe way

001: n/a

002: n/a

003: n/a

004: n/a

005: n/a

006: n/a

007: n/a

008: **I agree for sure. Knowledge is power, blah, blah, blah.**

009: n/a

010: n/a

011: n/a

012: n/a

013: n/a

014: n/a

015: n/a

016: n/a

017: n/a

018: n/a

1. Exercise can make the cartilage in my knee healthier

Alt: Exercise can make my joint healthier

Notes: Participants report neutral or unsure option being important for fact-based item

001: I wasn't sure if that meant that it was implying that cartilage regenerate or if exercise means that the cartilage is continuing to be used, but not abused.

002: I'm not quite sure if it can fix the cartilage

003: I don't know anything at all about the health in my knee. I cannot answer that question [LATER] I don't know how, but the answer is yes

004: n/a

005: I've been told I don't have any cartilage left, so I would say, um, disagree, but I don't even know if it's a true statement anyway, that it can, but I would answer it on the basis of my own experience.

A lot of people might think that cartilage every time you exercise, you know, you're wearing a, a bit away, um, from your cartilage. Um, whereas up as might think that, Oh, it's actually promoting, you know, healthy fluids around or whatever, um, and making it better. **This would be one where, because I don't know the answer, I'd be a bit torn about having it, I guess, or, um, actually just preferring an option that says, I don't know.**

006: n/a

007: n/a

008: n/a

009: n/a

010: n/a

011: I think I had a torn meniscus. **I don't even really understand what that is let alone what a cartilage is**

012: n/a

013: n/a

014: n/a

015: n/a

016: n/a

017: n/a

018: n/a

1. Doing more activity than normal can further damage my joint

Alt: Doing more than my usual amount of activity will further damage my joint

001: n/a

002: it's not saying what activity

003: n/a

004: n/a

005: it depends what normal is and that's what would be confusing. Um, so this would be a personal question. I would say that this would depend on individual circumstances

006: n/a

007: n/a

008: n/a

009: n/a

010: n/a

011: n/a

012: n/a

013: n/a

014: n/a

015: n/a

016: I don't agree with the statement, but that's a separate issue. **we don't know what the benchmark is**

017: n/a

018: n/a

1. Osteoarthritis must be rested

Alt: People with osteoarthritis should rest as much as possible

Alt: My joint should be rested as much as possible

001: I know the word rested is there, but **it doesn't say rested for how long**. **I've interpreted it as saying no more activity,** but that word rested needs. **If it was quantified, if it was must be initially rested, that might give a clearer answer.**

002: It can be, but not all the time

003: n/a

004: n/a

005: Maybe it should be “**must be rested in certain circumstances**”. Maybe if you’ve had an injury, um, might need to rest up…to give it a chance to heal”

006: n/a

007: **I just think that's a bit of a blanket statement**, Brian saying, osteoarthritis must be rested. Uh, what does that actually mean? **Does that mean you need to just rest all the time and for me that's certainly not the case.**

[participant suggested wording: “dealing with your osteoarthritis, uh, perhaps should incorporate some risking periods as well as your activity and your exercising.”

008: I mean, **you rest it at times don't you**. You rest it when you're in bed asleep, you can put the old recliner back but it's the word **must**, isn't it?

009: n/a

010: n/a

011: n/a

012: n/a

013: osteoarthritis, must be rested, I think **at times**. Yeah

014: n/a

015: n/a

016: **This statement hasn’t got a subject.** **It’s a nonsense statement**. my osteoarthritis, um, improves when I rest or something along those lines.

017: **could say “after exercise”?**

018: That one is a bit unclear, but **I presume it’s just a blanket statement that you must rest and not be active**, so I would put strongly disagree.

1. Slowly increasing my activity level over time increases how strong my body is, which allows me to do more activity than I previously could, without doing damage to my joint.

Alt: Gradually increasing my activity level over time increases how strong my body is, which allows me to do more activity than I previously could, without doing damage to my joint (remove period)

001: n/a

002: n/a

003: n/a

004: n/a

005: n/a

006: n/a

007: n/a

008: n/a

009: n/a

010: n/a

011: n/a

012: n/a

013: n/a

014: n/a

015: n/a

016: there's no real frame of reference here

017: n/a

018: n/a

1. Exercising contributes to the “wear and tear” of my osteoarthritis

Notes: Participants report neutral or unsure option being important for fact-based item

001: n/a

002: n/a

003: n/a

004: n/a

005: **I don’t know the answer**. I think what its saying is that the more exercise you do, naturally the wear and tear of your joints happens over time.

006: n/a

007: n/a

008: n/a

009: n/a

010: n/a

011: n/a

012: n/a

013: n/a

014: n/a

015: n/a

016: n/a

017: n/a

018: maybe that exercising because it doesn't necessarily have to be like exercise per se. It could just be, try and stand up and wash 10 loads of dishes at a time

1. My knowledge and beliefs about osteoarthritis and about exercise can influence how well I do

Alt: My understanding of osteoarthritis and exercise can influence my outcome

001: “**It's a little bit up in the air, ‘can influence, how well I do**’.” BRIAN: “Can you think of an example that would make sense for you?” 001: “can influence what activities I undertake”

002: n/a

003: n/a

004: n/a

005: if um, I think that exercise is bad for my knee will influence it because I won't do any, or if my knowledge and beliefs say that exercise is good, it will influence it because I will do it.

006: n/a

007: n/a

008: n/a

009: n/a

010: n/a

011: n/a

012: n/a

013: n/a

014: n/a

015: n/a

016: **I always worry when I say the word beliefs**. I need to come by a much stronger sense that they're going to do something about it. **I would use a word like knowledge instead of beliefs.**

017: n/a

018: n/a

1. There is little I can do to avoid flare-ups (of pain/swelling)

Alt: I know what to do to avoid flare-ups (of pain/swelling)

Notes: Participants report neutral or unsure option being important for fact-based item

001: again, **it's an, a negative**. … what if it was? **‘I know what to do to avoid flare ups’** or ‘I'm aware of what to do to avoid flare ups.’

002: n/a

003: n/a

004: n/a

005: n/a

006: n/a

007: n/a

008: n/a

009: n/a

010: n/a

011: n/a

012: n/a

013: n/a

014: n/a

015: n/a

016: n/a

017: n/a

018: I don’t have enough knowledge on that one, that I can remember anyway.

1. Flare-ups (of pain/swelling) are warning signs that I have injured or damaged my joint

Notes: Participants report neutral or unsure option being important for fact-based item

001: n/a

002: n/a

003: n/a

004: n/a

005: **If I had a choice to put ‘don’t know’… I’d probably put don’t know.**

006: n/a

007: n/a

008: n/a

009: n/a

010: n/a

011: n/a

012: n/a

013: n/a

014: n/a

015: n/a

016: n/a

017: n/a

018: n/a

1. My osteoarthritis cannot be improved with physical activity/exercise

Alt: My osteoarthritis **cannot** be improved with physical activity/exercise

001: n/a

002: n/a

003: n/a

004: n/a

005: n/a

006: n/a

007: n/a

008: n/a

009: n/a

010: n/a

011: n/a

012: n/a

013: when I saw this I read it or **didn't read it properly** {referring to the “cannot”}

014: n/a

015: n/a

016: n/a

017: n/a

018: n/a

1. Having an increased understanding of my body’s protective systems can help me to avoid or reduce flare-ups

Alt: Learning about my body’s protective systems can help me to avoid or reduce flare-ups

001: n/a

002: n/a

003: n/a

004: n/a

005: I think the question is straightforward, but **I’m not sure what you mean by** **protective systems**.

006: “any knowledge will help. Um, **I'm not sure what my body's** **protective systems** **as such are**. That's not a term that, um, you know, I've got a clinical understanding of” [agreed because ‘any knowledge will help’]

007: n/a

008: n/a

009: n/a

010: n/a

011: n/a

012: n/a

013: n/a

014: n/a

015: n/a

016: n/a Folks that perhaps don’t understand the concept…Maybe they'll understand the concept, but **maybe they need some more guidance on what protective systems** there are for the body

017: n/a

018: n/a

Are there additional questions you think this questionnaire should include?

**Assessment: Questions addressing diet were recommended by some participants.** Other additional question ideas are available through other common assessments including WOMAC

001: I'm wondering how I've got a background in how the body works, but I'm wondering if you asked, um, somebody without that background, where does that appreciate what the question was asking? And would they have the knowledge of how their body works? I guess you need that range of responses.

002: n/a

003: What activities are you doing? What activities are you capable of? Do you go to a group?

004: n/a

005: n/a

006: maybe, um, some more, um, direct questions about the treatment that I have had, um, Yeah. What might those be? maybe even just some opportunity at the end, too, to, um, discuss it or put down some thoughts about it.

007: n/a

008: n/a

009: n/a

010: How much time you spend sitting in the chair? can you get out of the bed at night-time without help to go to a toilet?

011: **Perhaps diet**

012: n/a

013: n/a

014: maybe, um, some more, um, direct questions about the treatment that I have had. maybe even just some opportunity at the end, to, um, discuss it or put down some thoughts about it.

015: n/a

016: something that is completely missing from this questionnaire is um, questions around our **eating habits**.

017: n/a

018: Well maybe you could add the question of what sort of physical activity do you think would be helpful. Something about the origins or the cause. There’s nothing about the origins.

Are there questions you think are irrelevant to you and should be removed?

**Assessment: Participant reflections do not warrant removing items.**

001: n/a

002: n/a

003: n/a

004: #3 It's just sort of the place I'm in what's it mean by the place i'm in, and what's it mean by the people I'm with how could they have an influence on my pain? I can't, yeah, that one doesn't make sense to me. #5 Well, I don't know that things in the world are going to have any effect on my knee.

005: n/a

006: n/a

007: [participant did report not understanding why some questions were included or valuable, but these were the same as those they didn’t understand]

008: n/a

009: n/a

010: n/a

011: n/a

012: n/a

013: n/a

014: n/a

015: (19: covered by some of the other questions) (5: It seems very very broad. Maybe you might want to get rid of “and the world”)

016: n/a

017: n/a

018: n/a
